# A Case of Nosocomial Outbreak of *Pantoea agglomerans* Related to Parenteral Nutrition Procedures

**DOI:** 10.3390/healthcare9060684

**Published:** 2021-06-07

**Authors:** Dora Mirtella, Piergiorgio Fedeli, Roberto Scendoni, Nunzia Cannovo, Mariano Cingolani

**Affiliations:** 1 Institute of Legal Medicine, University of Macerata, 62100 Macerata, Italy; roberto.scendoni@gmail.com (R.S.); mariano.cingolani@unimc.it (M.C.); 2 School of Law, Legal Medicine, University of Camerino, 62032 Camerino, Italy; piergiorgio.fedeli@unicam.it; 3 Ethic Committee, University of Naples, 80138 Naples, Italy; nunzia.cannovo@gmail.com

**Keywords:** nosocomial outbreak, *Pantoea agglomerans* sepsis, medical devices contamination, parenteral nutrition

## Abstract

*Pantoea agglomerans* is a Gram-negative bacterium that infrequently infects humans. Most reports involving it are about infections in soft tissues or bone/joint infections caused by contamination from soil or penetrating trauma by vegetation, such as thorns and splinters. More frequently, it is found as an opportunistic pathogen in immunocompromised patients. It is not rare to find reports of bacteremia and sepsis from contamination of intravenous fluid, parenteral nutrition, blood products and anesthetic agents—that is, through contamination of medical devices and products. We reported a bacterial infection epidemic occurred in 2011 in a hospital in middle Italy which involved different hospital wards; *Pantoea agglomerans* was isolated from blood cultures of all infected patients and the source of infection was identified in contaminated parenteral nutrition prepared from the local pharmacy.

## 1. Introduction

*Pantoea Agglomerans* is a facultative Gram-negative aerobic bacillus in the *Enterobacteriaceae* family. Formerly known as *Enterobacter agglomerans*, its classification was changed because it is present in a variety of geographic areas and multiple sources, including fecal material, plants and soil [1], both as a commensal and as a pathogen [2].

Infections in humans are not frequent, and mostly manifest in soft tissue or bone/joint caused by contamination from soil or penetrating trauma by vegetation, such as thorns and splinters [3,4,5,6,7,8,9] as well as cases of peritonitis in dialysis patients [10]. Vaiman et al. [11] found in a retrospective study that all nine patients with 1–2 months of ineffective treatment of post-traumatic wounds showed the presence of foreign bodies of plant origin infected with *Pantoea agglomerans*. Removal of the foreign bodies led to rapid healing of the wounds in 2–3 days. Septic arthritis or synovitis appears as common clinical outcome of exogenous infection with *Pantoea agglomerans* [4,5,12,13]. Others include endophthalmitis [14,15,16], acute peculiar bilateral dacryocystitis [17], and periostitis [18]. More frequently, it is found as an opportunistic pathogen in immunocompromised patients, with wound infections, sepsis and urinary tract infections, endocarditis in patients with valvular heart disease, pneumonia post-lung transplant [19], or in patients with chronic renal insufficiency.

It is not rare to find reports of bacteremia and sepsis from contamination of intravenous fluid [20], parenteral nutrition [21,22], blood products [23] and anesthetic agents [24]—that is, through contamination of medical devices and products. In fact, *Pantoea agglomerans* is one of the germs that caused the biggest nosocomial epidemic in the United States, in 1970–1971, with 378 cases of septicemia (of which 152 were only *Pantoea agglomerans*) and a 13.4% death rate, an epidemic caused by contamination of threaded screwcaps of bottles of parental solutions for intravenous injection [25].

The same contamination source was held responsible for an epidemic of nosocomial sepsis of *Pantoea agglomerans* and *Enterobacter cloacae* that involved 63 Greek newborns and babies with a mortality rate of 6.3% [20]. More recently, an outbreak of septicemia was described in Malaysia, with a high mortality rate (87.5%), involving newborns in an intensive care unit, caused by administration of parenteral nutrition infected by *Pantoea aggregans* [26].

Bicudo et al. reported a nosocomial epidemic of sepsis from *Pantoea agglomerans* in six pediatric patients in Brazil, caused by contaminated tubing used for intravenous hydration [27]. Also, in Brazil, Boszczowski et al. reported the outbreak of *Pantoea agglomerans* bacteremia in seven patients treated with hemodialysis or plasmapheresis, caused by contamination of an anticoagulant solution produced by the hospital pharmacy [28].

Cruz et al. reported 53 cases of *Pantoea agglomerans* infection in patients in a Houston pediatric hospital over the course of over six years [8]; the infections were mostly polymicrobial and the mortality rate was 5.7%. *Pantoea agglomerans* was mostly associated with penetration trauma by plant material and catheter bacteremia.

In Italy, Focà et al. described six cases of *Pantoea agglomerans* sepsis in a Calabrian University Polyclinic [29]. More recently, Izzo et al. reported 7 cases of septicemia in oncologic patients, caused by infected catheters [30].

Some *Pantoea agglomerans* infections associated with hospitalization were defined as sporadic-endogenous-spontaneous infections, as it was not possible to document the source of the germs, and most of the infections were related to a decline in the patient’s immune conditions and/or to hospital procedures [17,31,32,33,34].

In fact, *Pantoea agglomerans* infections have an opportunistic character and develop in immunocompromised patients. In most cases, the clinical course of the infection is mild and the administration of the proper antibiotic leads to complete recovery.

Looking at the risk factors described, *Pantoea agglomerans* infection can often be seen as a healthcare-associated infection, a concept that goes beyond nosocomial infections to embrace not only those contracted in hospitals (and there is an increased number of patients hospitalized in grave conditions, who are more susceptible to hospital infections). It also comprises those contracted in outpatient facilities, healthcare residences, and homes [35], where since the 1990s, healthcare services have also been provided.

The case presented by the authors is interesting because it adds to the scarce international case series and contributes to identify the moments of contamination of enteral nutrition bags. Therefore, the data made available for prevention purposes are useful, also with regard to the preparation of the healthcare personnel involved.

### Case Report

The case concerns a *Pantoea agglomerans* bacterial infection epidemic in 2011 in a hospital in central Italy, which at the time had a microbiology laboratory divided into two units, each managed by a different director.

On 31 August, in Bacteriological Laboratory 1, blood samples from two pediatric oncology patients (patients A and B) tested positive for Gram bacteria. After incubation, the bacteria was identified on 2 and 3 September, respectively, as *Pantoea agglomerans*.

The method used for identification of the microorganism present was MALDI (matrix-assisted laser desorption/ionization)/TOF (time-of-flight) mass-spectrometry (Bruker) [36,37]. MALDI is a soft ionization that involves a laser striking a matrix of small molecules to transform the analyte molecules into the gas phase without fragmenting or decomposing them. Some biomolecules are too large and can decompose when heated, and traditional techniques will fragment or destroy macromolecules. MALDI is appropriate to analyze biomolecules like peptides, lipids, saccharides, or other organic macromolecules.

On 4 and 5 September in the same laboratory, two other samples tested positive and were confirmed as the same bacteria on 7 and 8 September, respectively, in patients C and D, also in pediatric oncology. The director of Bacteriological Laboratory 1 asked the head nurse of the ward whether there was a correlation among the infections, and was told that only patients A and C had central venous catheters.

On 5 September, in Bacteriological Laboratory 2, the case of a positive blood culture for Gram bacteria was recorded for patient E from the General Surgery ward, and the patient’s ward was informed immediately by phone. On 6 September, the subculture of the positive bottle showed development of punctiform colonies, and in addition, five other samples from four patients tested positive for Gram bacteria: patients E, F (General Surgery), G (Internal Medicine) and H (Occupational Medicine).

Also, on 6 September, Bacteriology 1 recorded another sample that tested positive for Gram bacteria from patient I (Nephrology).

That same day, a director from the hospital’s Medical Management team, for unknown reasons, rather than contacting the Bacteriology 1 head directly, phoned the Bacteriology 2 director, who then informed the colleague.

On 7 September, Bacteriology 2 reported samples that tested positive for Gram bacteria for patients L (General Surgery), M (Emergency Surgery), and N (Internal Medicine), and on 8 September in the bloodwork of patients O (General Surgery) and P (Emergency Surgery). Spectrometric analyses were conducted on all the positive bottles and subcultures, and on 8 September the bacteria was identified as *Pantoea agglomerans*; the reports for patients E and G were drawn up and the results communicated to the wards as soon as they were ready.

The director of Bacteriology 1 informed the CIO (Hospital Committee on Infections) that all the infected patients were receiving parenteral nutrition, and so on 7 September, the CIO ordered its suspension and where possible, the removal of the central venous catheter, as well as examination of some nutrition bags in Bacteriology 1 and Bacteriology 2.

In these cases, the Total Parenteral Nutrition (TPN) bags were prepared “on demand” according to a physician’s prescription for a specific patient (“personalized”) [38] by the local Pharmacy Service as prescription magistral galenic preparations. Pharmacy technicians did these galenic preparations and the pharmacists prepared only pediatric bags, all of which proved to be free of infective phenomena.

On 8 September, Bacteriology 1 identified four bags out of six as positive for Gram- bacteria, with colonies similar to those of the blood cultures; in Bacteriology 2, four bags out of four tested positive.

To summarize, between 31 August and 8 September, Bacteriology 1 identified six patients who were positive for *Pantoea agglomerans*, while four out of the six kits for analysis of the parenteral nutrition bags analyzed at the same laboratory tested positive. Of note, patient **B** had two positive blood samples and a negative bag. On 9 September, the laboratory recorded the last positive test for G-bacteria, for patient **Q** (Vascular Surgery), which was reported on 13 September. 

Bacteriology 2 identified 13 patients with *Pantoea agglomerans*, from blood samples taken between 3 and 9 September, for 14 corresponding BACTEC bottles that tested positive in the period from 5 to 11 September, and four total parental nutrition bags for four patients delivered to the laboratory on 7 September. The last patients who tested positive on 9 September were patient **R** (Vascular Surgery), **S** and **T** (Emergency Surgery), and on 10 September, patient **U** (Internal Medicine). After 12 September, no other blood samples tested positive for *Pantoea agglomerans.*

The list of patients is shown in Table 1

The lists of parenteral nutrition bags analysed in laboratories Bacteriology1 and Bacteriology 2 are shown below in Table 2 and Table 3

Table 4 summarize the most relevant data for each patient.

To summarize, on the basis of the medical documents, the 19 patients who received TNP (Total parenteral nutrition) between 28 August and 6 September were a fairly heterogenous group in terms of gender, age, pathologies and wards. There were 10 males and nine females between the ages of 7 and 88, hospitalized in Pediatric Oncology (four patients: A, B, C, D), General Surgery (six patients: E, L, M, O, P, T), Occupational Medicine (two patients: F, H), Internal Medicine (three patients: G, N, U), Hematology (one patient: Q), Vascular Surgery (1 patient: R), Emergency Surgery (one patient: S), and Nephrology (one patient: I).

Two of the patients (G, U) received the parenteral nutrition at home; the bags were prepared by the Hospital’s Pharmacy Service.

It is certain that all 19 patients received TPN in the period of August–September 2011, and the clinical and laboratory diagnosis of sepsis due to *Pantoea agglomerans* is equally certain. In some of the cases (A, C, D, F, L, M, O, Q) the contents of the bags tested positive for *Pantoea agglomerans*. However, the documentation was not sufficient to ascertain whether the analyzed bags, prepared in the local hospital pharmacy specifically for each individual patient, were actually administered in the ward. Thus, it is not possible to establish with certainty for each individual patient a direct relationship between positive patient, positive bloodwork and positive bag.

Three of the 19 patients died (E, H, Q); the sepsis in the remaining 16 was treated with antibiotics without ill effects.

## 2. Discussion

This case clearly describes a healthcare-correlated infection, a concept that goes beyond nosocomial infections to embrace not only those contracted in hospitals but also those contracted in outpatient facilities, healthcare residences, and homes. Since the 1990s. there has been an increase in the number of patients hospitalized in grave conditions (and thus more vulnerable to hospital infections) and in the number of places for healthcare service outside the hospital, such as homes, healthcare residences and outpatient facilities. It is no coincidence that in this case, three of the patients were receiving healthcare at home.

The healthcare-correlated infection in this case was most probably due to by *Pantoea agglomerans* contamination of the parenteral nutrition bags. The protocol applied in laboratories Bacteriology 1 and Bacteriology 2 did not provide for genotyping.

It is well known that IV.-administered artificial nutrition can fully meet the nutritional needs of patients who are unable to eat or drink by natural means.

Parenteral nutrition bags contain a mixture of substances that can be:

- Provided exclusively by a pharmacy service, prepared “on demand” and “personalized” according to a physician’s prescription for a specific patient as prescription magistral galenic preparations; this was the case for the 19 patients;
- Provided upon request by a pharmacy service, as an official galenic preparation (prepared at an industrial facility), upon specific medical formulation according to current regulations;
- Provided upon request by a pharmacy service as a proprietary medicinal product.

The common factors among the 19 patients of this heterogeneous group were the nutrition bag prepared in the local pharmacy service, and the isolation in almost every case of *Pantoea agglomerans* over the course of about one week.

The identification of the nutrition bag as the source of contamination is not necessarily undermined by the fact that not all of the hospital patients on TPN in that period developed the infection. Contact with a pathogen does not always lead to disease, as various factors are involved, including as the immune status of the patient. Indeed, *Pantoea agglomerans* tends to cause nosocomial infections in immunodepressed, elderly and dialysis patients [39]. In addition, in some cases, a patient could have been infected but there might have been no laboratory evidence: naturally, not all the patients in the hospital had bloodwork done for *Pantoea agglomerans*. Also, it is entirely possible that not all the bags prepared in that period in that hospital were contaminated.

It is possible to exclude incorrect management of the central venous catheters or a patient’s particularly weak condition as causes of the contagion, given the limited period of time of the epidemic in a variety of wards, and above all the fact that there was one agent of infection common to all the patients who had received TPN in that period. 

Regarding the source of the infection, it is highly significant that infections occurred among the patients who received the personalized “on-demand” galenic TPN bags prepared by the pharmacy technicians, but not among those who received the pediatric TPN bags prepared exclusively by the pharmacists.

TPN bags must be prepared following both the Regulations for Proper Preparation indicated in the Official Pharmacopeia, and the Technical Standards for Galenic Parenteral Nutrition detailed by the Italian Society of Hospital Pharmacy [40], in a laboratory properly organized and equipped to avoid the risk of contamination. It is well known that during preparation, unacceptable concentrations of airborne particles and bacteria in the work area can cause contamination, as can incorrect handling of critical points, such as the point of withdrawal or injection, and the tubes of various kinds [41].

Studies have shown a positive correlation between particulate matter in the work area and microbial contamination. Thus, standard procedures have been developed to reduce this risk to a minimum [42,43]. One of the most important measures is the correct use of horizontal or vertical laminar flow hoods with class 100 HEPA filters [41].

According to the Official Pharmacopeia Regulations for Proper Preparation and the Technical Standards for Galenic Parenteral Nutrition detailed by the Italian Society of Hospital Pharmacy, sterile products, among them TPN bags, must be prepared in a class A room with specific standard filters for airborne particles and microbes. Given that the products used for parenteral nutrition do not present a biological risk for the operator, a horizontal laminar flow cabinet is a minimum requisite for protecting the final preparation from contamination.

In the case examined in this article, the laboratory had three horizontal laminar flow hoods that met the current norms and afforded a class A sterile environment in terms of airborne particle counts. The room was not classified. Two hoods had SIFRAMIX mixers and were used by the pharmacy technicians to prepare non-pediatric bags. The third station was reserved for use by the pharmacists to prepare the pediatric bags. Analysis of the data indicates that only the bags prepared by the technicians at the stations with the SIFRAMIX (Fresenius Kabi Italy Srl, Isola della Scala, Italy) mixer were contaminated by *Pantoea agglomerans*.

Regarding the moment of contamination, we can reasonably exclude its happening before the galenic preparation, because subsequent analyses of the ingredients used to prepare the bags demonstrated that they were sterile. Also, it would be very difficult to posit that the contamination happened after the preparation, and thus two possibilities remain:

- Contamination by staff who brought the bags to the patients.
- Incorrect administration of the TPN, for example, failure to sterilize the injection site or mistakes in changing the IV tube or in handling the central venous catheters.

These hypotheses are also weak given the pluriform manifestation of the contagion in a variety wards as well as in the homes of patients, especially considering that in home care, hospital staff do not administer the TPN. Also, the fact that the bags were contaminated only with *Pantoea agglomerans* does not support either of these hypotheses.

Another hypothesis to be excluded as unlikely is retrograde contamination, given the high number of contaminated bags, the fact that the contamination was found in a variety of wards, and the system of administration through 15 to 70 cm-long tubes, as the flow of the preparation would inhibit the flow of material containing the microorganism toward the bag.

Considering all of these factors, it is highly likely that the bags were contaminated during the on-demand personalized galenic preparation by the pharmacy technicians. This is further supported by the documentation for a patient extraneous to the case, who received but was not administered a bag that later tested positive for *Pantoea agglomerans* contamination.

In fact, though the laboratory had three hoods with identical characteristics, which, when examined as part of the investigation, proved to function well and to provide sterile conditions, only the bags produced under the hoods with SIFRAMIX mixers by the pharmaceutical technicians were contaminated. The bags for pediatric patients were prepared by the pharmacist under the third hood, so the contamination did not arise from malfunction of the hoods.

The closed SIFRAMIX system inside a class A laminar hood affords sterile conditions if the technician observes the regulations and standards. Specifically, the technician should load the device with the components for the TPN bags and connect them with a kit that should be changed daily. The loading of the SIFRAMIX is a key passage and if done incorrectly, may allow contamination of the final product.

In this case, lack of documentation about the status and training level of the technicians hinders an understanding of factors that might explain incorrect conduct by the technician. However, there is documentation that the staff members of the pharmacy service responsible for preparing the bags failed to observe not only the Regulations for Proper Preparation.

In addition, the technicians did not compile the proper worksheets that should accompany the preparation of the TPN bags. These sheets should detail the lot number and expiration date of the constituents of the bag so that the substances used for the preparation can be traced. Also, the label that the technicians applied to the finished product did not include the details required by the Regulations for Proper Preparation.

While the absence or precariousness of a formal quality system, and in particular, the documentation it entails, made this case more complex and to some degree, made the retrospective reconstruction of what happened less complete. The data available points quite clearly to the contamination of the TPN bags was caused by the incorrect practices of the technicians.

Sterility can be assured only by strict observance of the regulations for proper preparation, by dedicated work areas, by appropriate equipment, by qualified staff, by procedures of cleaning and disinfection, by the cycle of sterilization used, by the aseptic techniques used, and by microbiological monitoring of the work areas.

According to Italian law [42], magistral and officinal preparations must pass sterility testing and a test for bacterial endotoxins, as prescribed in Italian Pharmacopeia.

While the implementation of a quality management system demands time and resources, in the interests of protecting the health of citizens, it is essential that every healthcare structure have one. By now, this should be the cultural patrimony and operative mode of all healthcare structures.

The macroscopic lapses in communication in this case doubtlessly contributed to the delay in sharing data about the spread of the infection. On 6 September, with nine patients infected by the same bacteria, there was no evidence of contact between the directors of the two laboratories, or between them and the hospital management. The failures in communication may have been favored by the fact of having two laboratories and two directors, but very probably were exacerbated and perpetuated by a mode of communication that was contorted, to say the least. In this case, only the modest aggressiveness *Pantoea agglomerans* spared most patients from worse outcomes. The deaths of patients E, H and Q can be attributed to the fact that their health conditions were seriously compromised even before the infection. Patient Q died about two months after E and H, and for this patient, bloodwork was done at the time of death. Thus, bloodwork was only done for this one patient out of the three who died.

## 3. Conclusions

*Pantoea agglomerans* is a rare pathogen, but has been reported to cause severe infections in immunocompromised.

This report it adds to the scarce medical literature and propose to contribute to identify the source and moment of contamination, in patients hospitalized, in order to improve preventive measures.

## Figures and Tables

**Table 1:** Patients listed by gender, age, disease, ward and the laboratory that tested their blood samples.

Patient	Gender	Age	Disease	Ward	Laboratory
A	M	17	ALL	Ped. Oncology	B1
B	F	7	ALL	Ped. Oncology	B1
C	M	11	DLBCL	Ped. Oncology	B1
D	F	8	ALL/s. Down	Ped. Oncology	B1
E	M	Not known	intestinal adenocarcinoma	Gen. Surgery	B2
F	F	69	gastric adenocarcinoma	Occupational Medicine	B2
G	F	72	malabsorption	Internal Medicine	B2
H	F	68	colorectal adenocarcinoma	Occupational Medicine	B2
I	M	68	m. Crohn/colon adenoma	Nephrology	B1
L	M	71	pancreas carcinoma	Gen. Surgery	B2
M	M	77	diverticular disease	Gen. Surgery	B2
N	F	77	aortomesenteric bypass	Internal Medicine	B2
O	M	88	colorectal adenocarcinoma	Gen. Surgery	B2
P	M	39	m. Crohn/intestinal subocclusion	Gen. Surgery	B2
Q	F	27	pneumonia post transplant	Hematology	B1
R	M	76	aneurisma abdominal aorta	Vascular Surgery	B1
S	F	49	bacterial peritonitis	Emergency Surgery	B2
T	F	69	colon cancer/uterine cancer	Gen. Surgery	B2
U	M	39	cancer cachexia	Internal Medicine	B2

Gender: M = male; F = female. Laboratory: B1 = Bacteriology 1; B2 = Bacteriology 2. ALL Acute Lymphoblastic Leukemia. DLBCL = diffuse large B cell lymphoma.

**Table 2:** List of parenteral nutrition bags analyzed by Bacteriology 1.

Patient	Ward	Appearance of the Bag	Date Accessed	Date of Report
A	Ped. Oncology	milky	unknown	Unknown
C	Ped. Oncology	clear	7 September	9 September
D	Ped. Oncology	clear	7 September	10 September
Q	Hematology	milky	7 September	9 September

**Table 3:** List of parenteral nutrition bags analyzed by Bacteriology 2.

Patient	Ward	Appearance of the Bag	Date Accessed	Date of Report
F	Gen. surgery	milky	6 September	8 September
M	Emergency surgery	clear pale straw yellow	6 September	8 September
L	Gen. Surgery	milky	6 September	8 September
O	Gen. Surgery	milky	6 September	8 September

**Table 4:** Patients, date of administration of Total Parenteral Nutrition in the hospital (marked with diagonals) or at home (H), date the bloodwork tested positive (blood +), date of the report (report+), date the bag tested positive (bag+), outcome, and laboratory that performed the analyses (B1 = Bacteriology 1, B2 = Bacteriology 2).

Patients	28/08	29/08	30/08	31/08	1/09	2/09	3/09	4/09	5/09	6/09	Blood+	Report+	Bag+	Outcome	Lab
**A**	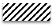	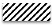	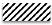	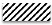	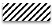	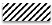			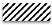	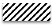	31.08	02.09	?		B1
**B**			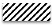	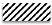		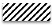		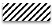		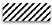	01.09	03.09			B1
**C**		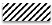		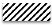	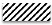	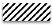	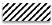	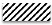	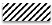	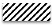	01.09	05.09	07.09		B1
**D**					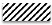	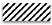	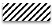	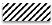		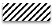	05.09	07.09	07.09		B1
**E**		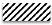	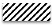	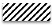	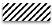	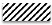	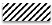	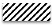			05.09	08.09		Died 4.09	B2
**F**						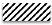	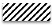	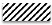	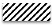	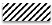	06.09	13.09	07.09		B2
**G**	**H**	**H**	**H**	**H**	**H**	**H**	**H**	**H**	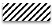	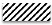	06.09	08.09			B2
**H**							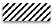	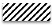	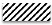	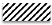	06.09	13.09		Died 7.09	B2
**I**				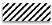	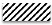	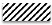	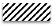		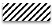		06.09	09.09			B1
**L**						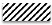	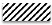	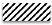	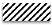	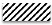	06.09	13.09	07.09		B2
**M**									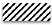	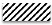	06.09	13.09	07.09		B2
**N**	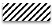	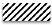	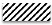		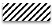	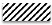		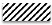	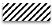	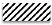	6-7.09	13.09			B2
**O**									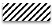	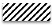	08.09	13.09	07.09		B2
**P**										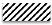	08.09	13.09			B2
**Q**									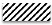	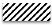	09.09	12.09	07.09	Died 21.10	B1
**R**									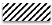	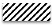	09.09	12.09			B1
**S**									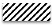	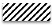	09.09	13.09			B2
**T**				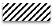	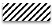	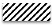	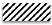	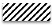	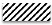	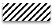	09.09	13.09			B2
**U**	**H**	**H**	**H**	**H**	**H**	**H**	**H**	**H**	**H**	**H**	10.09	13.09			B2

## Data Availability

No new data were created or analyzed in this study. Data sharing is not applicable to this article.
